# MRI reveals scoliosis as a silent driver of lumbar facet joint degeneration in aging Ecuadorian adults

**DOI:** 10.1038/s41598-025-04994-2

**Published:** 2025-06-05

**Authors:** Pablo Cueva-Medina, Jorge Silva-Hidalgo, Fabricio González-Andrade

**Affiliations:** 1 Universidad San Francisco de Quito USFQ, Colegio Ciencias de la Salud, Escuela de Especialidades Médicas, calle Diego de Robles s/n y Pampite, 170901 Quito, Ecuador; 2 Universidad Tecnológica Indoamérica, Facultad de Ciencias de la Salud, Dirección de Posgrados, calle Machala y Sabanilla, 170301 Quito, Ecuador

**Keywords:** Lumbar facet arthropathy, Scoliosis, Chronic low back pain, Cobb angle, Magnetic resonance imaging, Joint degeneration, Anatomy, Diseases

## Abstract

Lumbar facet arthropathy (LFA) is a degenerative condition of the facet joints that contributes significantly to chronic low back pain (CLBP) yet remains underrecognized in standard diagnostic protocols. While aging and disc degeneration are established factors in the progression of LFA, the potential role of spinal deformities such as scoliosis has not been thoroughly investigated. This cross-sectional study examined the association between scoliosis severity and LFA in a non-surgical population of 284 Ecuadorian adults aged 30 to 80 years. Participants were classified by Cobb angle into normal, mild, moderate, and severe scoliosis categories and underwent MRI to assess facet joint degeneration at the L3–L4, L4–L5, and L5–S1 levels. Findings revealed that moderate scoliosis significantly increased the odds of severe LFA across all lumbar levels, particularly at L3–L4 (OR = 6.72) and L5–S1 (OR = 5.57). Mild scoliosis also posed a notable risk, with a threefold increase at L4–L5 and a twofold increase at L5–S1. Additionally, degeneration was more severe on the concave side of the scoliotic curve. Older age and female sex were also independently associated with more advanced degeneration. These results suggest that scoliosis, even in its mild forms, plays a significant role in the development and progression of LFA. The findings highlight the importance of including facet joint evaluation in routine scoliosis assessment and CLBP workups, particularly using MRI. Early identification of individuals at risk could inform preventive strategies and reduce long-term disability related to degenerative spinal disease.

## Introduction

Chronic low back pain (CLBP) is one of the most prevalent musculoskeletal disorders worldwide, representing a major cause of disability and healthcare burden. Among its multifactorial causes, lumbar facet arthropathy (LFA) is a common but often underdiagnosed contributor to axial back pain in adults^1^. The lumbar facet joints are synovial articulations located at the posterior vertebral column that, together with intervertebral discs, form a three-joint complex responsible for maintaining spinal stability by distributing axial loads and limiting excessive motion^1^.

Anatomically, lumbar facet joints are oriented in the coronal plane, allowing effective transmission of compressive and torsional forces^2^. However, facet tropism—defined as asymmetry in facet joint orientation where one facet is more sagittally aligned than its contralateral counterpart—can result in altered biomechanics and increased vulnerability to degeneration^3^. Degenerative changes often begin with intervertebral disc deterioration, leading to compensatory overloading of the facet joints and accelerated osteoarthritic changes^4^.

LFA is histologically characterized by cartilage erosion, subchondral sclerosis, joint space narrowing, and osteophyte formation^5^. These features may appear as early as adolescence, although radiological signs typically become more evident after the age of 40^6,7^. The facet joints are innervated by the medial branches of the dorsal rami, and their degeneration is often associated with complex pain patterns, including referred and radicular pain, making clinical diagnosis challenging^8,9^.

Imaging techniques such as computed tomography (CT) and magnetic resonance imaging (MRI) are valuable for evaluating LFA. MRI provides detailed visualization of joint morphology, including capsular thickening, joint effusion, subchondral cysts, and cartilage loss, enabling the identification and grading of LFA severity^10,11^.

Beyond intrinsic joint degeneration, spinal deformities—particularly scoliosis—have been implicated in the exacerbation of degenerative changes in the lumbar spine. The Cobb angle, introduced by Cobb in 1948, remains the gold standard for assessing spinal curvature^12^. Adult degenerative scoliosis (ADS) is defined as a spinal curvature of ≥ 10° in the coronal plane in skeletally mature individuals and is often caused by asymmetric degeneration of discs and facet joints^13^. It may develop de novo or as a progression of adolescent idiopathic scoliosis^14^.

Biomechanically, scoliosis alters spinal load distribution, increasing stress on the concave side of the curve, where compression forces are greater. This asymmetric loading pattern is believed to accelerate degeneration of the facet joints and intervertebral discs^15,16^. However, despite the plausible pathophysiological link, the precise relationship between scoliosis severity and LFA progression remains poorly elucidated in clinical studies^17^.

To date, most research on scoliosis and facet degeneration has focused on surgical populations or high-income countries, with limited data available from non-surgical community-based cohorts in Latin America. Ecuador, with its demographic diversity and increasing access to diagnostic imaging, offers a unique setting to explore this association in an underrepresented population.

This study aims to assess the association between lumbar facet arthropathy and scoliosis in a sample of Ecuadorian adults. Specifically, we assessed how scoliosis severity—measured using the Cobb angle—correlates with LFA severity at the L3-L4, L4-L5, and L5-S1 levels, while accounting for potential confounders such as age and sex.

## Methods

This cross-sectional, observational, and epidemiological study was designed to investigate the relationship between lumbar facet arthropathy (LFA) and scoliosis severity in Ecuadorian adults. The study protocol received ethical approval from the Research Ethics Committee of the Universidad San Francisco de Quito (Approval No. IE02-E223-2021-CEISH-USFQ), and all participants provided informed consent.

A total of 284 adults between the ages of 30 and 80 were included, consisting of 142 individuals diagnosed with scoliosis and 142 without scoliosis. None of the participants had a history of spinal surgery. All subjects underwent anteroposterior lumbar spine radiographs to evaluate spinal alignment, and magnetic resonance imaging (MRI) to assess the severity of facet joint degeneration.

Scoliosis was defined and categorized based on the Cobb angle, measured on standing frontal radiographs following standard radiological methods^12^. Participants were grouped as follows: normal alignment (< 10°), mild scoliosis (10°–24°), moderate scoliosis (25°–44°), and severe scoliosis (≥ 45°). Given that only one individual presented with severe scoliosis (≥ 45°), this category was not included in subgroup analyses due to insufficient statistical power.

MRI was used to assess LFA at three lumbar levels: L3–L4, L4–L5, and L5–S1. Facet joint degeneration was graded based on standard radiographic features, including joint space narrowing, subchondral sclerosis, osteophyte formation, and hypertrophy, as described in validated imaging criteria^10,11^. Imaging evaluations were performed using data from the AXXISCAN PACS (Picture Archiving and Communication System) database in Quito, Ecuador. All images were reviewed by two experienced radiologists, and any discrepancies in interpretation were resolved by consensus.

Demographic information such as age and sex, as well as clinical characteristics including pain patterns and curve concavity, were collected from the patients’ medical records. Scoliosis concavity (left or right) was recorded to explore possible asymmetrical degeneration patterns.

For cases where nerve root involvement was suspected on MRI, the Weishaupt classification was employed. This system classifies neural compression into four grades: grade 0 (no contact with the nerve root), grade 1 (contact without displacement), grade 2 (displacement without compression), and grade 3 (definite nerve root compression)^18^.

Statistical analysis was conducted using IBM SPSS version 28. Descriptive statistics included frequencies and percentages for categorical variables, and medians with interquartile ranges for continuous variables. The Shapiro–Wilk test was applied to evaluate the normality of age distribution. Differences in continuous variables between scoliosis groups were assessed using the Mann–Whitney U test, and the Kruskal–Wallis test was used for comparisons among multiple LFA severity categories. Chi-square or Fisher’s exact tests were used for categorical variables. A multivariate logistic regression analysis was performed to identify independent predictors of severe LFA at each spinal level, using scoliosis severity, age, sex, and curve concavity as input variables. Statistical significance was set at *p* < 0.05. To adjust for the increased risk of Type I error due to multiple comparisons, Bonferroni correction was applied.

## Results

**Fig. 1:**
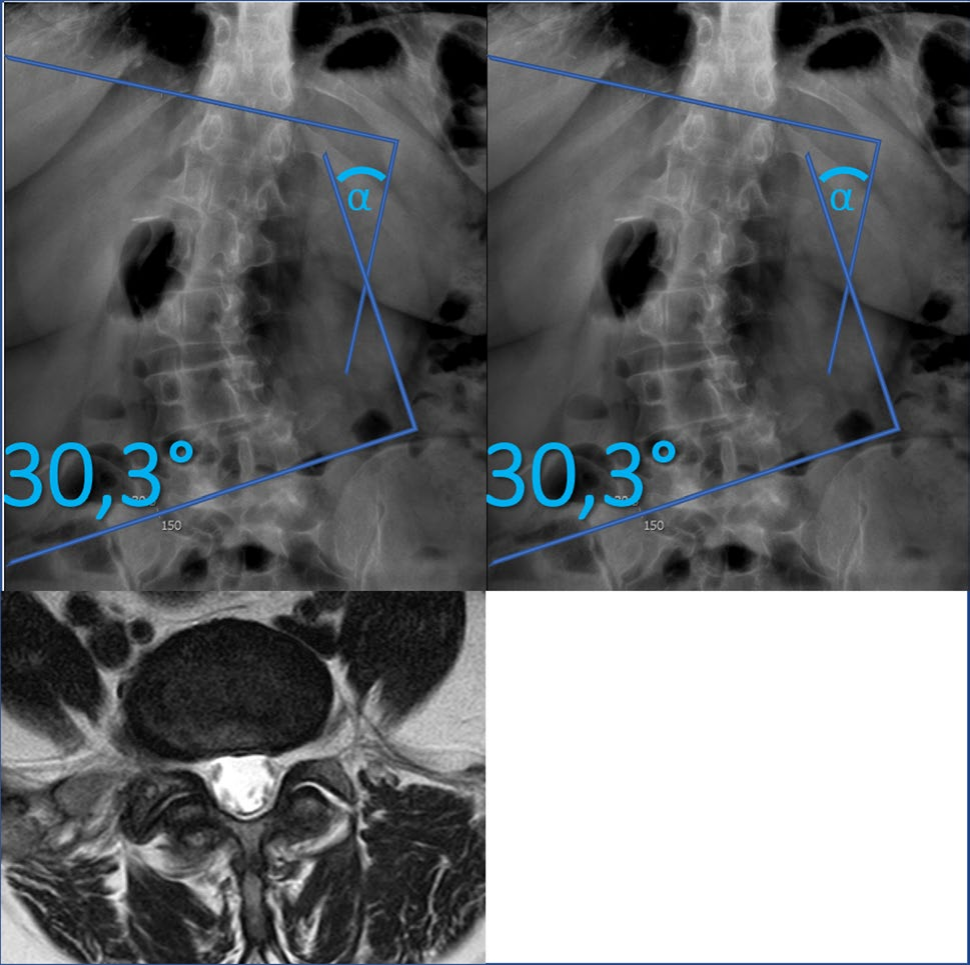
Moderate scoliosis with a Cobb angle of 30.3° plus signs of severe LFA due to marginal osteophytes, sclerosis hypertrophy, and a cyst on the right facet (AP X-ray, sagittal and axial T2-wighted MRI).

**Fig. 2:**
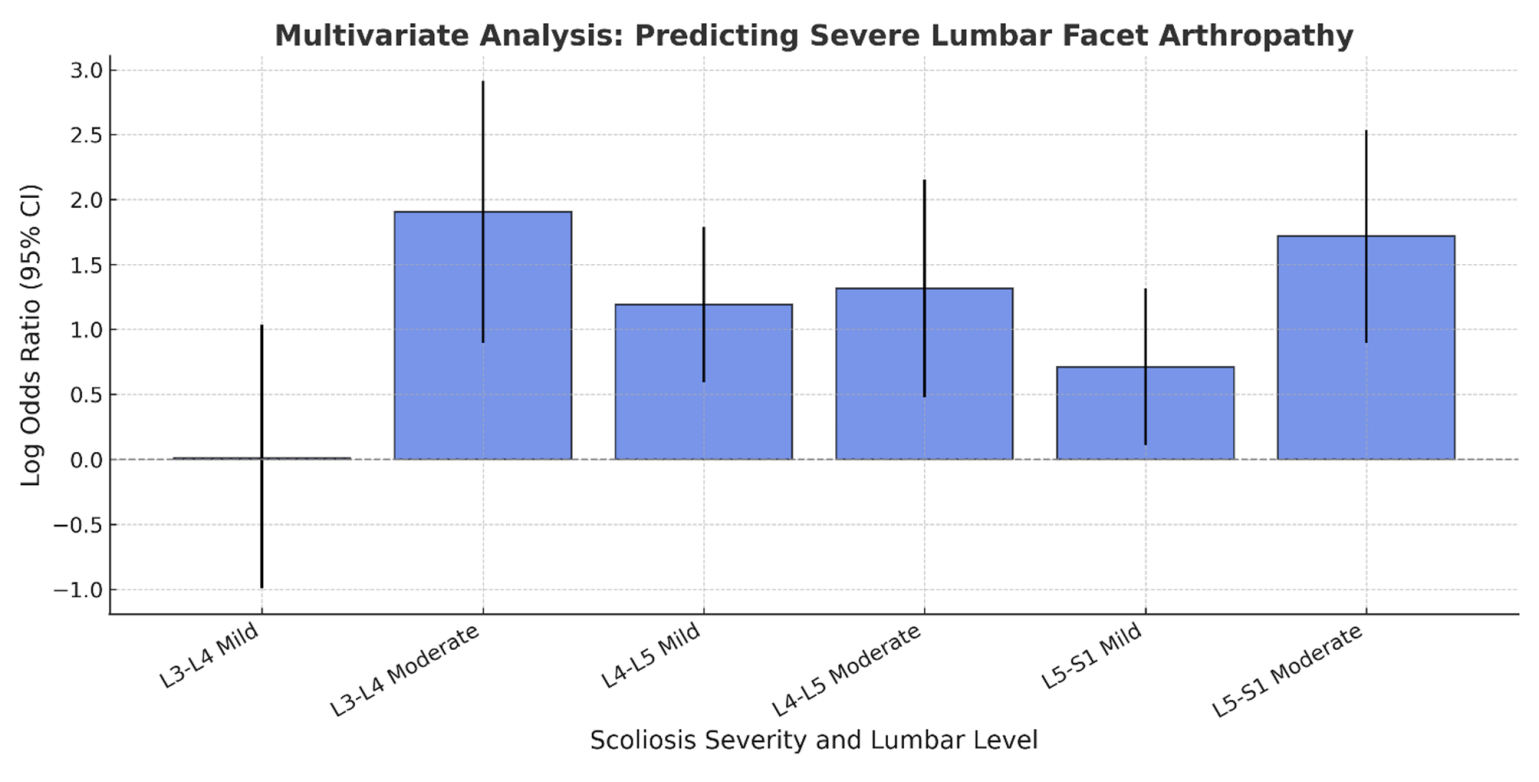
It is a bar chart visualizing odds ratios for predicting severe lumbar facet arthropathy (LFA) at different lumbar levels based on scoliosis severity. The y-axis represents the log-transformed odds ratio with 95% confidence intervals (CI). The x-axis represents different scoliosis conditions at L3-L4, L4-L5, and L5-S1. The blue bars indicate the increased risk of severe LFA, with higher bars representing greater risk. The dashed horizontal line at 0 represents an odds ratio of 1 (no effect).

Table 1 summarizes clinical and imaging characteristics by scoliosis status. Tables 2 and 3, and 4 present LFA severity at L3–L4, L4–L5, and L5–S1, respectively, showing associations with age, sex, Cobb angle, and pain characteristics. Table 5 displays logistic regression results, identifying moderate scoliosis as a strong predictor of severe LFA across all levels. Figure 1 shows moderate scoliosis (Cobb angle 30.3°) with severe LFA at L4–L5, including osteophytes, sclerosis, hypertrophy, and a right-sided facet cyst (on AP X-ray and T2-weighted MRI). Figure 2 visualizes odds ratios: moderate scoliosis increases severe LFA risk at L3–L4 (OR = 6.72) and L5–S1 (OR = 5.57); mild scoliosis elevates risk at L4–L5 (OR = 3.29) and L5–S1 (OR = 2.04). These data reinforce scoliosis—especially moderate curves—as a key risk factor for LFA, highlighting the importance of early detection in clinical evaluation.

**Table 1:** Distribution of patients by presence or absence of scoliosis according to clinical characteristics and imaging findings.

Clinical characteristics and imaging findings	Total	Scoliosis		*p*-value
		Present	Absent	
Age (median (IQR))1/	57 (45–67)	63 (54–69)	50 (42–64)	< 0.001*
Sex (n (%))2/				
Male	103 (36.27)	42 (29.58)^a^	61 (42.96)^b^	0.019*
Female	181 (63.73)	100 (70.42)^a^	81 (57.04)^b^	
Low back pain (n (%))2/	202 (71.13)	101 (71.13)	101 (71.13)	1.000
Pain location (n (%))2/				
Low back pain	82 (36.12)	44 (34.65)	38 (38)	0.104
Lumbosciatica	84 (37)	42 (33.07)	42 (42)	
Other	61 (26.87)	41 (32.28)	20 (20)	
Pain chronicity (n (%))2/				
Subacute	24 (12.37)	7 (7.07)	17 (17.89)	0.073
Acute	9 (4.64)	5 (5.05)	4 (4.21)	
Chronic	161 (82.99)	87 (87.88)	74 (77.89)	
Cobb angle (n (%))2/				
Normal / Scoliotic attitude	142 (50)	0 (0)	142 (100)	< 0.001*
Mild scoliosis	109 (38.38)	109 (76.76)	0 (0)	
Moderate scoliosis	32 (11.27)	32 (22.54)	0 (0)	
Severe scoliosis	1 (0.35)	1 (0.7)	0 (0)	
Artropatía facetaria lumbar L3-L4 (n (%))^2/^				
Normal	65 (22.89)	22 (15.49)^a^	43 (30.28)^b^	0.012*
Leve	121 (42.61)	62 (43.66)^a^	59 (41.55)^a^	
Moderada	71 (25)	40 (28.17)^a^	31 (21.83)^a^	
Severa	27 (9.51)	18 (12.68)^a^	9 (6.34)^a^	
Lumbar facet arthropathy L3-L4 (n (%))2/				
Normal	12 (4.23)	0 (0)^a^	12 (8.45)^b^	< 0.001*
Mild	59 (20.77)	18 (12.68)^a^	41 (28.87)^b^	
Moderate	136 (47.89)	69 (48.59)^a^	67 (47.18)^a^	
Severe	77 (27.11)	55 (38.73)^a^	22 (15.49)^b^	
Lumbar facet arthropathy L5-S1 (n (%))2/				
Normal	29 (10.21)	5 (3.52)^a^	24 (16.9)^b^	< 0.001*
Mild	111 (39.08)	45 (31.69)^a^	66 (46.48)^b^	
Moderate	71 (25)	43 (30.28)^a^	28 (19.72)^b^	
Severe	73 (25.7)	49 (34.51)^a^	24 (16.9)^b^	
Greater severity of facet arthropathy (n (%))^2/^				
L3-L4	23 (8.65)	10 (7.35)	13 (10)	0.728
L4-L5	152 (57.14)	78 (57.35)	74 (56.92)	
L5-S1	91 (34.21)	48 (35.29)	43 (33.08)	
Concavity side (n (%))^2/^				
Right	130 (45.77)	75 (52.82)^a^	55 (38.73)^b^	< 0.001*
Left	104 (36.62)	63 (44.37)^a^	41 (28.87)^b^	
None	50 (17.61)	4 (2.82)^a^	46 (32.39)^b^	
Greater concavity severity (n (%))^2/^				
Concave	74 (26.06)	46 (32.39)^a^	28 (19.72)^b^	< 0.001*
Convex	62 (21.83)	46 (32.39)^a^	16 (11.27)^b^	
Indistinct	148 (52.11)	50 (35.21)^a^	98 (69.01)^b^	

IQR = Interquartile Range; * significant differences. Different superscripts indicate categories that differ. 1/Mann Whitney. 2/Chi-square test.

Source: AXXISCAN. own elaboration.

**Table 2:** Distribution of patients by degree of AFL L3-L4 according to clinical characteristics and imaging findings.

Clinical features and imaging findings	Lumbar facet arthropathy degree L3-L4				*p*-value
	Normal	Mild	Moderate	Severe	
Age (median (IQR))^1/^	47 (37–56)^a^	51 (43–65)^a^	65 (60–72)^b^	67 (56–76)^b^	< 0.001*
Sex (n (%))^2/^					
Male	25 (38.46)	47 (38.84)	26 (36.62)	5 (18.52)	0.244
Female	40 (61.54)	74 (61.16)	45 (63.38)	22 (81.48)	
Low back pain (n (%))^2/^	46 (70.77)	83 (68.6)	52 (73.24)	21 (77.78)	0.773
Location of pain (n (%))^2/^					
Low back pain	24 (50)^a^	31. (34.07)^a.b^	16 (25)^b^	11. (45.83)^a,b^	0.033*
Lumbosciatica	15 (31.25)^a^	39 (42.86)^a^	26 (40.63)^a^	4 (16.67)^a^	
Other	9 (18.75)^a^	21 (23.08)^a^	22 (34.38)^a^	9 (37.5)^a^	
Chronicity of pain (n (%))^2/^					
Subacute	5 (11.36)	7 (8.97)	8 (15.69)	4 (19.05)	0.202
Acute	5 (11.36)	2 (2.56)	2 (3.92)	0 (0)	
Chronic	34 (77.27)	69 (88.46)	41 (80.39)	17 (80.95)	
Cobb angle (n (%))^2/^					
Normal/Scoliotic attitude	43 (66.15)^a^	59. (48.76)^a.b^	31. (43.66)^a.b^	9 (33.33)^b^	< 0.001*
Mild scoliosis	21 (32.31)^a^	48 (39.67)^a^	33 (46.48)^a^	7 (25.93)^a^	
Moderate scoliosis	1 (1.54)^a^	14 (11.57)^a^	7 (9.86)^a^	10 (37.04)^b^	
Severe scoliosis	0 (0)^a^	0 (0)^a^	0 (0)^a^	1 (3.7)^a^	
Concavity side (n (%))^2/^					
Right	26 (40)^a^	60 (49.59)^a^	32 (45.07)^a^	12 (44.44)^a^	0.001*
Left	16 (24.62)^a^	51 (42.15)^a^	25 (35.21)^a^	12 (44.44)^a^	
None	23 (35.38)^a^	10 (8.26)^b^	14. (19.72)^a.b^	3. (11.11)^a.b^	
Greater concavity severity (n (%))^2/^					
Concave	10 (15.38)	38 (31.4)	21 (29.58)	5 (18.52)	0.139
Convex	15 (23.08)	21 (17.36)	17 (23.94)	9 (33.33)	
Indistinct	40 (61.54)	62 (51.24)	33 (46.48)	13 (48.15)	

IQR = Interquartile Range; * significant differences. Different superscripts indicate a different degree of arthropathy. 1/Kruskal Wallis. 2/ Chi-square test.

Source: AXXISCAN. own elaboration.

**Table 3:** Distribution of patients by degree of lumbar facet arthropathy L4-L5 according to clinical characteristics and imaging findings.

Clinical features and imaging findings	Lumbar facet arthropathy degree L4-L5				*p*-value
	Normal	Mild	Moderate	Severe	
Age (median (IQR))^1/^	42 (36–45)a	47 (37–55)^a^	58 (47–65)^b^	66 (60–75)^b^	< 0.001*
Sex (n (%))^2/^					
Male	6 (50)	25 (42.37)	43 (31.62)	29 (37.66)	0.352
Female	6 (50)	34 (57.63)	93 (68.38)	48 (62.34)	
Low back pain (n (%))^2/^	6 (50)	41 (69.49)	97 (71.32)	58 (75.32)	0.341
Location of pain (n (%))^2/^					
Low back pain	5 (83.33)	15 (34.09)^b^	43 (39.45)^b^	19 (27.94)^b^	0.121
Lumbosciatica	1 (16.67)	17 (38.64)	41 (37.61)	25 (36.76)	
Other	0 (0)	12 (27.27)	25 (22.94)	24 (35.29)	
Chronicity of pain (n (%))^2/^					
Subacute	1 (16.67)	5 (13.16)	11 (11.83)	7 (12.28)	0.345
Acute	1 (16.67)	4 (10.53)	2 (2.15)	2 (3.51)	
Chronic	4 (66.67)	29 (76.32)	80 (86.02)	48 (84.21)	
Cobb angle (n (%))^2/^					
Normal/Scoliotic attitude	12 (100)^a^	41 (69.49)^a^	67 (49.26)^b^	22(28.57)^b^	< 0.001*
Mild scoliosis	0 (0)^a^	17 (28.81)^a^	51. (37.5)^a.b^	41 (53.25)^b^	
Moderate scoliosis	0 (0)^a^	1 (1.69)^a^	18 (13.24)^b^	13 (16.88)b	
Severe scoliosis	0 (0)^a^	0 (0)^a^	0 (0)^a^	1 (1.3)^a^	
Concavity side (n (%))^2/^					
Right	7 (58.33)	22 (37.29)	62 (45.59)	39 (50.65)	0.052
Left	2 (16.67)	18 (30.51)	55 (40.44)	29 (37.66)	
None	3 (25)	19 (32.2)	19 (13.97)	9 (11.69)	
Greater concavity severity (n (%))^2/^					
Concave	0 (0)^a,b^	8 (13.56)^a^	45 (33.09)^b^	21 (27.27)^a,b^	0.006*
Convex	1 (8.33)^a^	12 (20.34)^a^	31 (22.79)^a^	18 (23.38)^a^	
Indistinct	11 (91.67)^a^	39. (66.1)^a,b^	60 (44.12)^b^	38 (49.35)^b^	

IQR = Interquartile Range; * significant differences. Different superscripts indicate a different degree of arthropathy. 1/Kruskal Wallis. 2/ Chi-square test.

Source: AXXISCAN. own elaboration.

**Table 4:** Distribution of patients by degree of lumbar facet arthropathy L5-S1 according to clinical characteristics and imaging findings.

Clinical features and imaging findings	Lumbar facet arthropathy degree L5-S1				*p*-value
	Normal	Mild	Moderate	Severe	
Age (median (IQR))^1/^	40 (35–50)^a^	51 (43–64)^a^	60 (50–68)^b^	64 (59–71)^b^	< 0.001*
Sex (n (%))^2/^					
Male	14 (48.28)	38 (34.23)	28 (39.44)	23 (31.51)	0.387
Female	15 (51.72)	73 (65.77)	43 (60.56)	50 (68.49)	
Low back pain (n (%))^2/^	13 (44.83)^a^	82 (73.87)^b^	50 (70.42)^b^	57 (78.08)^b^	0.008*
Location of pain (n (%))^2/^					
Low back pain	6 (42.86)	32 (36.36)	17 (27.87)	27 (42.19)	0.197
Lumbosciatica	5 (35.71)	38 (43.18)	20 (32.79)	21 (32.81)	
Other	3 (21.43)	18 (20.45)	24 (39.34)	16 (25)	
Chronicity of pain (n (%))^2/^					
Subacute	2 (16.67)	9 (11.54)	8 (16.67)	5 (8.93)	0.342
Acute	1 (8.33)	4 (5.13)	4 (8.33)	0 (0)	
Chronic	9 (75)	65 (83.33)	36 (75)	51 (91.07)	
Cobb angle (n (%))^2/^					
Normal/Scoliotic attitude	24 (82.76)^a^	66. (59.46)^a,b^	28 (39.44)^a,b^	24 (32.88)^b^	< 0.001*
Mild scoliosis	5 (17.24)^a^	37 (33.33)^a,b^	35 (49.3)^b^	32 (43.84)^b^	
Moderate scoliosis	0 (0)^a^	8 (7.21)^a^	7 (9.86)^a.b^	17 (23.29)^b^	
Severe scoliosis	0 (0)	0 (0)	1 (1.41)	0 (0)	
Concavity side (n (%))^2/^					
Right	13 (44.83)	48 (43.24)	33 (46.48)	36 (49.32)	0.513
Left	8 (27.59)	40 (36.04)	29 (40.85)	27 (36.99)	
None	8 (27.59)	23 (20.72)	9 (12.68)	10 (13.7)	
Greater concavity severity (n (%))^2/^					
Concave	5 (17.24)^a^	24 (21.62)^a^	22 (30.99)^a^	23 (31.51)^a^	< 0.001*
Convex	2 (6.9)^a^	16 (14.41)^a^	20 (28.17)^a,b^	24 (32.88)^b^	
Indistinct	22 (75.86)^a^	71 (63.96)^a^	29 (40.85)^b^	26 (35.62)^b^	

IQR = Interquartile Range; * significant differences. Different superscripts indicate a different degree of arthropathy. 1/Kruskal Wallis. 2/ Chi-square test.

Source: AXXISCAN. own elaboration.

**Table 5:** Multivariate relationship to predict the severe degree of lumbar facet arthropathy L3-L4, L4-L5 and L5-S1 based on the Cobb angle.

	B	Wald	*p*-value	OR	IC-OR 95%	
					Lower	Upper
L3-L4						
Mild scoliosis	0.014	0.001	0.978	1.010	0.37	2.81
Moderate scoliosis	1.905	13.737	< 0.001*	6.72**	2.45	18.39
L4-L5						
Mild scoliosis	1.191	15.26	< 0.001*	3.29**	1.81	5.98
Moderate scoliosis	1.317	9.460	0.002*	3.73**	1.61	8.64
L5-S1						
Mild scoliosis	0.71	5.41	0.020*	2.04**	1.12	3.73
Moderate scoliosis	1.72	16.80	< 0.001*	5.57**	2.45	12.67

The study included a total of 284 Ecuadorian adults, equally divided into scoliosis (*n* = 142) and non-scoliosis (*n* = 142) groups. The median age of the entire sample was 57 years, with statistically significant differences observed between groups. Participants with scoliosis had a higher median age of 63 years compared to 50 years in the non-scoliosis group (*p* < 0.001). Female predominance was noted overall (63.73%), with a higher proportion of women in the scoliosis group (70.42%) than in the non-scoliosis group (57.04%) (*p* = 0.019). Conversely, men were more frequently represented in the non-scoliosis group.

Pain symptoms were reported by 71.13% of participants, of whom 82.99% experienced chronic pain. The most reported symptoms included lumbar and sciatic pain (37.01%), followed by isolated low back pain (36.12%) and other pain patterns (26.87%). Based on Cobb angle measurements, 50% of participants had normal spinal curvature (< 10°), while 38.38% had mild scoliosis, 11.27% had moderate scoliosis, and only one participant (0.35%) exhibited severe scoliosis (≥ 45°). Due to the small size of the latter subgroup, statistical analysis involving the severe scoliosis category was not feasible.

Analysis of MRI findings revealed significant associations between scoliosis presence and LFA severity at all three lumbar levels. At the L3–L4 level, normal facet joint morphology was present in only 15.49% of individuals with scoliosis, compared to 30.28% in those without. At L4–L5, normal morphology was absent (0%) in the scoliosis group, while 8.45% of non-scoliosis individuals displayed normal joints. Mild LFA was more prevalent among non-scoliosis individuals (28.87%) compared to scoliosis cases (12.68%), whereas severe LFA was substantially higher in the scoliosis group (38.73% vs. 15.49%). At L5–S1, normal joint morphology was found in 3.52% of scoliosis cases and 16.90% of non-scoliosis cases. Severe LFA was again more frequent in scoliosis patients (34.51%) compared to those without scoliosis (16.90%). Across the population, the L4–L5 segment exhibited the highest prevalence of severe LFA (57.14%), followed by L5–S1 (34.21%) and L3–L4 (8.65%).

Curve concavity was also significantly associated with LFA severity. Among scoliosis patients, 52.82% had right-sided concavity and 44.37% had left-sided concavity. In contrast, concavity was absent in 32.39% of individuals without scoliosis but only in 2.32% of those with scoliosis (*p* < 0.001). Severe LFA was more frequent on the concave side of the spinal curve.

Logistic regression analysis demonstrated that scoliosis severity was an independent predictor of severe LFA. At L3–L4, moderate scoliosis increased the odds of severe LFA by 6.72 times (*p* < 0.001; OR = 6.72; 95% CI: 2.45–18.39). Mild scoliosis was not significantly associated with severe degeneration at this level. At L4–L5, both mild and moderate scoliosis were significant predictors. Mild scoliosis was associated with a 3.29-fold increase in severe LFA risk (*p* < 0.001; OR = 3.29; 95% CI: 1.81–5.98), and moderate scoliosis increased the risk by 3.73 times (*p* = 0.002; OR = 3.73; 95% CI: 1.61–8.64). At L5–S1, mild scoliosis doubled the risk (*p* = 0.020; OR = 2.04; 95% CI: 1.12–3.73), while moderate scoliosis increased the risk more significantly (*p* < 0.001; OR = 5.57; 95% CI: 2.45–12.67).

In summary, the data revealed a strong association between scoliosis severity and LFA progression, with the highest risk of severe degeneration observed in individuals with moderate scoliosis at L3–L4 and L5–S1. Mild scoliosis also conferred significant risk, particularly at the L4–L5 and L5–S1 levels. Additionally, older age and female sex were correlated with increased LFA severity, and degeneration was more pronounced on the concave side of the spinal curvature. These findings emphasize the importance of including scoliosis assessment in the diagnostic evaluation of LFA.

## Discussion

This study provides robust evidence of a strong association between scoliosis severity and lumbar facet arthropathy (LFA), particularly at the L3–L4, L4–L5, and L5–S1 levels. Moderate scoliosis emerged as the most significant predictor of severe LFA, with odds ratios exceeding fivefold in some lumbar segments. These results support the hypothesis that spinal misalignment introduces biomechanical stress that accelerates facet joint degeneration. The correlation was strongest at the L3–L4 and L5–S1 levels, suggesting that curvature-induced mechanical asymmetry has site-specific effects on facet joint health.

Notably, even mild scoliosis was associated with significantly increased odds of severe LFA at L4–L5 and L5–S1. These findings challenge the traditional assumption that mild scoliosis is clinically insignificant in adults. Given that L4–L5 and L5–S1 are critical load-bearing segments of the lumbar spine, it is plausible that even small curvatures result in pathological stress concentrations at these levels. This biomechanical stress may compound age-related degeneration and contribute to early-onset or rapidly progressing facet arthropathy.

Age and sex also emerged as important modifiers of LFA severity. Participants with moderate or severe facet degeneration were significantly older, supporting well-established evidence that aging is a major risk factor for joint degeneration due to cumulative mechanical wear, inflammation, and cartilage breakdown^5,6^. The study also identified a clear female predominance among individuals with scoliosis and severe LFA. This finding aligns with existing literature suggesting that postmenopausal hormonal changes, particularly decreased estrogen levels, contribute to spinal joint deterioration through reduced cartilage integrity and increased inflammatory activity^7^.

The association between scoliosis concavity and LFA severity further strengthens the biomechanical interpretation of these findings. Facet degeneration was more severe on the concave side of the scoliotic curve, where mechanical compression and load concentration are greater^16^. This observation aligns with prior studies describing asymmetric joint degeneration patterns in adult degenerative scoliosis and reinforces the role of load imbalance in the pathogenesis of facet arthropathy^15^.

The use of MRI in this study allowed detailed and reliable grading of facet joint changes, contributing to the strength of the diagnostic assessment. Unlike plain radiography, MRI can detect early cartilaginous and subchondral changes, providing a more comprehensive view of facet joint pathology^10,11^. Incorporating scoliosis screening and facet joint evaluation into standard imaging protocols for chronic low back pain (CLBP) may improve early detection of individuals at risk for degenerative spinal disease.

Nonetheless, this study has several limitations. Its cross-sectional design precludes causal inference, and longitudinal studies are necessary to confirm the progression of LFA in relation to scoliosis over time. Additionally, although the overall sample size was sufficient, the small number of individuals with severe scoliosis limited statistical analysis in that subgroup. Future research should investigate the impact of therapeutic interventions such as physical therapy, bracing, or targeted rehabilitation in patients with mild to moderate scoliosis, especially those with early signs of LFA.

## Conclusion

This study demonstrates a clear and quantifiable association between scoliosis severity and lumbar facet arthropathy (LFA) in a non-surgical Ecuadorian adult population. Moderate scoliosis significantly increased the risk of severe facet degeneration at all lumbar levels, particularly at L3–L4 and L5–S1, while even mild scoliosis was associated with elevated risk at L4–L5 and L5–S1. These findings indicate that spinal curvature, even within traditionally “mild” ranges, should not be considered biomechanically benign. The results also confirm that age, female sex, and curve concavity are relevant factors influencing LFA severity. Given the observed pattern of asymmetric degeneration, integrating facet joint evaluation into scoliosis assessment protocols may enhance early identification of high-risk patients and guide more targeted, preventive spinal care.

## Data Availability

The datasets used and/or analysed during the current study available from the corresponding author on reasonable request.

## Abbreviations

ADS: Adult degenerative scoliosis
CLBP: Chronic low back pain
CT: Computed tomography
CI: Confidence interval
IC-OR: Confidence interval for odds ratio
LFA: Lumbar facet arthropathy
MRI: Magnetic resonance imaging
OR: Odds ratio
PACS: Picture archiving and communication system
SPSS: Statistical package for the social sciences
p-value: Probability value (for statistical significance)
